# Back to Acid Soil Fields: The Citrate Transporter SbMATE Is a Major Asset for Sustainable Grain Yield for Sorghum Cultivated on Acid Soils

**DOI:** 10.1534/g3.115.025791

**Published:** 2015-12-17

**Authors:** Geraldo Carvalho, Robert Eugene Schaffert, Marcos Malosetti, Joao Herbert Moreira Viana, Cicero Bezerra Menezes, Lidianne Assis Silva, Claudia Teixeira Guimaraes, Antonio Marcos Coelho, Leon V. Kochian, Fred A. van Eeuwijk, Jurandir Vieira Magalhaes

**Affiliations:** * Embrapa Maize and Sorghum, Sete Lagoas, Minas Gerais, 35701-970 Brazil; † Biometris, Wageningen University and Research Center, Wageningen, 6700AC The Netherlands; ‡ Robert W. Holley Center of Agriculture and Health, USDA-ARS, Cornell University, Ithaca, New York 14853

**Keywords:** Al tolerance, QTL mapping, *Sorghum bicolor*, *Alt_SB_*, field trials

## Abstract

Aluminum (Al) toxicity damages plant roots and limits crop production on acid soils, which comprise up to 50% of the world’s arable lands. A major Al tolerance locus on chromosome 3, *Alt_SB_*, controls aluminum tolerance in sorghum [*Sorghum bicolor* (L.) Moench] via SbMATE, an Al-activated plasma membrane transporter that mediates Al exclusion from sensitive regions in the root apex. As is the case with other known Al tolerance genes, *SbMATE* was cloned based on studies conducted under controlled environmental conditions, in nutrient solution. Therefore, its impact on grain yield on acid soils remains undetermined. To determine the real world impact of *SbMATE*, multi-trait quantitative trait loci (QTL) mapping in hydroponics, and, in the field, revealed a large-effect QTL colocalized with the Al tolerance locus *Alt_SB_*, where *SbMATE* lies, conferring a 0.6 ton ha^–1^ grain yield increase on acid soils. A second QTL for Al tolerance in hydroponics, where the positive allele was also donated by the Al tolerant parent, SC283, was found on chromosome 9, indicating the presence of distinct Al tolerance genes in the sorghum genome, or genes acting in the *SbMATE* pathway leading to Al-activated citrate release. There was no yield penalty for *Alt_SB_*, consistent with the highly localized Al regulated *SbMATE* expression in the root tip, and Al-dependent transport activity. A female effect of 0.5 ton ha^–1^ independently demonstrated the effectiveness of *Alt_SB_* in hybrids. Al tolerance conferred by *Alt_SB_* is thus an indispensable asset for sorghum production and food security on acid soils, many of which are located in developing countries.

At soil pH values below 5.0, aluminum (Al) is solubilized as the rhizotoxic cation Al^3+^, which damages plant roots and inhibits root growth, reducing water and nutrient uptake, and crop yields (Kochian *et al.* 2004). Approximately 50% of the potentially arable lands worldwide are acidic (von Uexküll and Mutert 1995), mostly in the tropics and subtropics. Agriculture in regions such as sub-Saharan Africa is strongly constrained by Al toxicity (Doumbia *et al.* 1993, 1998), and Al tolerance can thus help liberate an enormous food production potential exactly where food security is most tenuous.

Aluminum-induced organic acid release protects the sensitive growing zone in the root apex by chelating Al as nontoxic complexes (Ma *et al.* 2001; Ryan *et al.* 2001), leading to sustained root growth in nutrient solution containing Al^3+^ (Kochian *et al.* 2004). Major Al tolerance genes belonging to the Aluminum-Activated Malate Transporter (ALMT), and the Multidrug and Toxic Compound Extrusion (MATE) families were cloned in wheat (*TaALMT1*) (Sasaki *et al.* 2004), sorghum (*SbMATE*) (Magalhaes *et al.* 2007), and barley (*HvAACT1*) (Furukawa *et al.* 2007) via hydroponic-based assessment of Al tolerance.

In sorghum, *SbMATE* underlies *Alt_SB_*, a major Al tolerance locus mapped to sorghum chromosome 3, and explains a large proportion of the phenotypic variation for Al tolerance assessed in hydroponics (Magalhaes *et al.* 2004, 2007). SbMATE is an Al-regulated (for both gene expression and protein function) root citrate transporter that acts to exclude Al from sensitive regions in the root apex, thus conferring Al tolerance (Magalhaes *et al.* 2007). *TaALMT1*, the major wheat Al tolerance gene, is located on the long arm of wheat chromosome 4D (Raman *et al.* 2005), and encodes an Al-activated malate transporter (Sasaki *et al.* 2004) that likely underlies the major wheat Al tolerance locus, *Alt_BH_* (Riede and Anderson 1996). Homologs of those two genes have also been identified in other plant species, including rye (Collins *et al.* 2008; Yokosho *et al.* 2010), maize (Maron *et al.* 2010), wheat (Ryan *et al.* 2009), Arabidopsis (Hoekenga *et al.* 2006; Liu *et al.* 2009), rape (Ligaba *et al.* 2006), rice (Yokosho *et al.* 2011), rice bean (Liu *et al.* 2013), barley (Zhou *et al.* 2013), and soybean (Liang *et al.* 2013).

There is an apparent agreement between sustained root growth under Al stress in hydroponics and yield traits assessed on acid soils (Reid *et al.* 1971; Duncan *et al.* 1983; Culvenor *et al.* 1986; Furlani *et al.* 1991; Baier *et al.* 1995). However, because Al tolerance genes were not isolated based primarily on field assessments of Al tolerance, their breeding value in enhancing grain yield on acid soils remains undetermined. Consequently, although the knowledge of the molecular basis of crop Al tolerance offers an unprecedented repertoire of novel strategies for achieving sustainable yields under Al stress, this potential remains highly underutilized for crop breeding.

In the present study, Al tolerance assessed in hydroponics and in field conditions was explained largely by a major QTL colocalized with *Alt_SB_* on chromosome 3, which increased grain yield by 0.6 ton ha^–1^ without any detectable penalty in the absence of Al toxicity. Evidence for a second, novel Al QTL on chromosome 9 based on Al-inhibition of root growth was obtained, suggesting the presence of either a second, distinct Al tolerance gene in the sorghum genome or a gene (or genes) acting with *SbMATE*, leading to Al-activated citrate release. An independent assessment of the *Alt_SB_* effect using isogenic hybrids for the Al tolerance locus yielded a female effect of 0.5 ton ha^–1^, arising from one copy of the tolerant allele at *Alt_SB_*. Our results show that *Alt_SB_* is a major asset for sustainable sorghum production on acid, Al toxic soils, and should be used to enhance food security under Al toxicity on acid soils prevalent in developing countries in the tropics and subtropics.

## Materials and Methods

### Plant material

#### Recombinant inbred line population:

A recombinant inbred line (RIL) population consisting of 90 F_7:8_ progeny derived from a cross between BR007, an Al-sensitive breeding line from the Embrapa Maize and Sorghum breeding program, and SC283, a highly Al-tolerant sorghum line belonging to the guinea race (Magalhaes *et al.* 2004), was developed by the single seed descent method (Johnson and Bernard 1962). Only 3-dwarf types were used to minimize the confounding effect of plant height variation on grain yield.

#### Development of isogenic hybrid stocks:

The effect of *Alt_SB_* on grain yield was also assessed in sorghum hybrids by comparing grain yield in homozygous sensitive genotype designated as (tt), heterozygous (Tt), or homozygous tolerant (TT) hybrids for *Alt_SB_*. Isogenic hybrids were developed to allow comparison of *Alt_SB_* alleles from different donors within a homogeneous genetic background.

##### NIL development:

ATF13B and ATF14B are sister nonrestorer lines in A1 cytoplasm derived by selfing a single F_5_ plant heterozygous for *Alt_SB_* derived from BR007 (tt) × SC283 (TT). Homozygous sensitive and tolerant progeny were selected by progeny testing for Al tolerance and self-pollinated for at least seven generations, giving rise to ATF13B (Al sensitive) and ATF14B (Al tolerant) lines. ATF13B and ATF14B were backcrossed to a source of A1 cytoplasmic male sterility (BR007A) for six generations to produce ATF13A and ATF14A. ATF13A (tt) and ATF14A (TT) are thus near isogenic lines (NILs) fixed for alternative *Alt_SB_* alleles derived from the Al-sensitive and Al-tolerant parents of our RIL population, BR007 and SC283, respectively.

BR012R is an Al-sensitive restorer line (tt), whereas SC549, CMS225, and SC566 are Al-tolerant lines (TT) in increasing level of tolerance (Caniato *et al.* 2007, 2011). NILs fixed for *Alt_SB_* were developed by introgressing from each Al tolerant donor the respective *Alt_SB_* alleles into the BR012R background. The SC566-NIL (BC_3_F_3_) (Melo *et al.* 2013), and the SC549- and CMS225- NILs (BC_6_F_3:8_) are fixed for the donor alleles at *Alt_SB_*. The NILs with the genetic background belonging to BR012 were designated here as BR012(SC566), BR012(SC549) and BR012(CMS225).

##### Hybrid production:

Each of the four isogenic male restorer (R) lines were crossed with the two female (A) lines to generate the hybrids H1 to H8 shown in Supporting Information, Table S1, which depicts the different male and female parents, and each of the derived isogenic hybrids.

Grain yield differences between H1(tt) and H2(Tt) resulted from substituting one Al-sensitive allele from BR007 with one *Alt_SB_* allele donated by the Al-tolerant parent, SC283. The comparisons H1(tt) *vs.* H3(Tt) *vs.* H4 (TT) gives the effect in grain yield of one *Alt_SB_* allele donated by SC566 (H3) and two Al tolerant alleles, with one donated by SC283 and one donated by SC566 (H4). Similar comparisons for the SC549 and CMS225 alleles were obtained by contrasting H1(tt) *vs.* H5(Tt) *vs.* H6 (TT), and H1(tt) *vs.* H7(Tt) *vs.* H8 (TT), respectively, with the homozygous tolerant hybrid being always formed by one *Alt_SB_* allele from SC283 in combination with another tolerant allele, from SC549 or CMS225.

### Hydroponic evaluation of Al tolerance

Aluminum-inhibition of seminal root growth was used to quantify Al tolerance, using the basal nutrient solution described in Magnavaca *et al.* (1987). Al tolerance was assessed in nutrient solution containing 0 or 148 μM Al added as AlK(SO_4_)_2_⋅12H_2_O, which correspond to {0} and {27}μM Al^3+^ activities, respectively (brackets indicate free Al^3+^ activity, as estimated with the speciation software GEOCHEM-PC) (Parker *et al.* 1995). The 27 µM Al^3+^ activity has been shown to adequately differentiate SC283 and BR007 for Al tolerance (Magalhaes *et al.* 2004, 2007; Caniato *et al.* 2007, 2011). Seeds were first scarified with sterile sand to break seed dormancy, surface-sterilized with NaOCl 0.1% for 8 min under continuous agitation, and rinsed eight times with 50 ml of 18MΩ H_2_O. Seeds were subsequently transferred to germination paper rolls moistened in 18MΩ H_2_O, and germination was allowed to proceed for 4 d in a growth chamber with a light intensity of 550 µmol photons m^–2^ sec^–1^, and a 12-hr photoperiod with 27 ± 3° day and 20 ± 3° night average temperature.

The seminal root from each seedling was then inserted through the mesh bottoms of polyethylene cups, covered with black beads, and placed in plastic tubs with 8 liters of nutrient solution under continuous aeration. A 24-hr acclimation period was subsequently applied in nutrient solution lacking Al. The *i*nitial *l*ength of each seminal root in *c*ontrol solution (*il_c_*) was measured after the acclimation period, and the *f*inal *l*ength in control solution (*fl_c_*) was recorded after 5 d in nutrient solution lacking Al. Similarly, *i*nitial root *l*ength (*il_Al_*) and *f*inal root *l*ength (*fl_Al_*) under *Al*-stress were recorded immediately after the acclimation period, and after 5 d of Al exposure, respectively. Accordingly, a control root growth rate (*crgr_5d_*) was obtained as [*crgr_5d_* = (*fl_c_*) – (*il_c_*)], and the root growth rate under Al exposure (*Alrgr_5d_*) was then calculated as [*Alrgr_5d_* = (*fl_Al_*) – (*il_Al_*)]. Relative percent values of net root growth (RNRG) inhibition were obtained as [RNRG = (*Alrgr_5d_* / *crgr_5d_*) × 100]. Each experiment was arranged as a randomized complete block design with two replications and seven plants per replication. The Al tolerant and sensitive NILs, ATF10B and ATF8B, which were derived from the same parents of the RIL population (Magalhaes *et al.* 2007), were included as controls.

### Field trials

#### Development of a field site for Al tolerance phenotyping:

The Al tolerance phenotyping sites were developed at the Embrapa Maize and Sorghum research station in Sete Lagoas (Minas Gerais, Brazil) at 19°27’57” latitude south, and 44°14’49” longitude west, and 766.73 m altitude. Two field sites under a typical Haplustox were characterized for aluminum saturation in the 0–20 cm, and the 20–40 cm soil layers. In the control site, dolomitic limestone containing ∼33% CaO and ∼10% MgO was applied and incorporated in the 0–20 cm soil layer to achieve low exchangeable Al saturation. No limestone was applied to the Al toxicity site. Two months after liming, the area was divided into rectangular 10 × 10 m and 5 × 5 m grids for the control and Al toxicity site, respectively. Topsoil (0–20 cm) and subsoil (20–40 cm) soil compound samples were collected on the square grid. Accordingly, a total of 51 and 169 points were sampled for each depth interval in the control and Al toxicity site, respectively. Three subsamples located in a maximum radius of 1 m from each sampling grid point were collected, and the compound samples were used for soil analysis. The measurements of soil exchangeable aluminum (Al^+3^) and exchangeable cations (Ca^+2^, Mg^+2^) were performed with KCl 1 mol liter^–1^ as extractor (Donagema *et al.* 2011) using inductively-coupled argon plasma (ICP) emission spectrometry. Percent values of Al saturation were calculated as the ratio of exchangeable Al^3+^ divided by the sum of basic cations plus Al^+3^.

#### Field phenotyping of Al tolerance:

Field experiments were conducted from April to September 2007 for the RIL population, and from January to April 2013 for the isogenic hybrids in soils with 2% and 56% Al saturation. For the RIL population, each plot consisted of two 5-m rows, with 0.45 m between rows, and eight plants m^–1^. In the hybrid experiment, each plot consisted of four 5-m rows, with 0.45 m between rows, and eight plants m^–1^ in a row. In this case, only the two center rows were harvested and considered for subsequent analysis. For both trials, fertilization consisted of 300 kg ha^–1^ of 8-28-16 (NPK) applied at planting, and 160 kg ha^–1^ of urea applied 30 d thereafter. The RIL experiments consisted of an incomplete block design with three replicates, and 10 incomplete blocks in each of the two environments, defined as control site (2% Al saturation), and Al toxicity site (56% Al saturation). A total of 100 genotypes in each replication included 90 RILs and the two parents, BR007 and SC283, with the Al tolerant parent, SC283, repeated nine times. The hybrid experiments consisted of a split plot experiment with the Al treatment (Al *vs.* control) applied at the whole plot level, and the hybrids applied at the sub plot level. At the whole plot level, a completely randomized design was assumed to be applicable with the two treatments (Al, control), each repeated four times. Each of the eight hybrid combinations (Table S1) was randomly allocated to a sub plot within each of the eight whole plots. In total, 64 experimental plots were used: four replicates × two whole plot treatments × eight sub plot treatments. Sorghum grain was manually harvested based on the average physiological grain maturity of the population, which occurred approximately 130 d after seed emergence. Total grain yield in ton ha^–1^ was estimated based on average grain weight per plot.

### Statistical analysis of phenotypic data

All statistical analyses described below were performed using GenStat 17 (VSN International 2014), except the map construction, which was done by the Onemap (Margarido *et al.* 2007) software.

#### Hydroponic data:

A randomized complete block design was used for assessing Al tolerance in hydroponics. Data collected was logarithmically transformed to the base 10 before subsequent analysis (henceforth any reference to RNRG will correspond to log-transformed data). A mixed model taking genotype random was fitted as follows; response = block + genotype + error. The variance components for the genotypic and error variance, $\sigma_{g}^{2}$ and $\sigma_{e}^{2}$, respectively, were used to calculate the heritability at a line mean basis; *h*^2^ = $\frac{\sigma_{g}^{2}}{\sigma_{g}^{2}+\sigma_{e}^{2}/r}$, where *r* corresponds to the number of blocks, which was two. Subsequently, genotype was fitted as a fixed term in order to obtain best linear unbiased estimators (BLUE) for later use in QTL mapping.

#### Field data:

For the RIL experiment, a multi-environment mixed model analysis was fitted as follows: response = treatment + replicate within treatment + incomplete block within replicate + genotype + error. In this model, the incomplete block, the genotype and the error term were assumed to be normally distributed with treatment dependent variances, *i.e.*, the variances were different between Al and control. For the genotype term, we assumed a covariance between the treatments, *i.e.*, between Al and control. Heritability at a line mean basis, *h*^2^, was estimated for Al and control treatment as *h*^2^ = $\frac{\sigma_{g}^{2}}{\sigma_{g}^{2}+\sigma_{e}^{2}/r}$, where $\sigma_{g}^{2}\;\mathrm{and}\;\sigma_{e}^{2}\;$ are the genotypic and error variances, respectively, and *r* corresponds to the number of replicates, which was three. Another mixed model analysis was done with genotypes fixed to produce genotypic BLUEs for multi-trait QTL analysis.

Split plot analysis of variance was performed for the hybrids experiment to test the effects of substituting alleles at the *Alt_SB_* locus. The effect of treatment, Al *vs.* control, was tested against the whole plot error, while all other effects were tested against the subplot error.

### Marker analysis and linkage map construction

The same sorghum linkage map as developed and described by Sabadin *et al.* (2012) was used in this study. Accordingly, a total of 344 markers consisting of 255 Diversity Arrays Technology (DArT), 83 SSRs, five Sequence-Tagged Site (STS), and one RFLP marker, were used for map construction. The RFLP marker, ISU52, was genotyped as described in Magalhaes *et al.* (2004), while genotyping with the STS markers, CTG29 and M181, was as described in Caniato *et al.* (2007). The STS fluorescent markers DG1, EM1, M1672, and M9612 were described in Sabadin *et al.* (2012).

### QTL analysis

The genotypic means for yield as observed in the field under Al and control conditions, and the RNGR genotypic means as obtained on the hydroponics medium were analyzed together in a multi-trait QTL analysis. A multi-trait QTL analysis facilitates the assessment of pleiotropic action of QTL and will have higher power for QTL detection. For our QTL analysis we followed a mixed model-based protocol as described for multi-trait and multi-environment QTL analyses by (Boer *et al.* 2007; Malosetti *et al.* 2008, 2013; Alimi *et al.* 2013). The protocol consisted of an initial round of simple interval mapping (Lander and Botstein 1989) that was followed by one or more rounds of composite interval mapping (Jansen and Stam 1994; Zeng 1994). From the last round of composite interval mapping, a candidate set of QTL was obtained, from which possibly redundant QTL were removed by a backward elimination procedure, with a final set of QTL as end result.

In the simple interval mapping round, the following mixed model was fitted along the genome for the three responses jointly; responses = QTL + polygenic effects + errors, with an unstructured variance covariance model imposed on the polygenic effects, *i.e.*, polygenic variances were trait specific, and polygenic correlations were unique for each pair of traits. The QTL allele substitution effects were fixed and trait specific. In the simple interval mapping scan, genomic positions were tested one by one for QTL presence by Wald tests. A multiple test correction was applied according to Li and Ji (2005). In composite interval mapping, potential QTL elsewhere in the genome were included as cofactors; responses = cofactors + QTL + polygenic effects + errors. The final multi-trait multi-QTL model was of the form responses = QTL + polygenic effects + errors. The amount of genetic variance explained by a QTL was estimated from the drop in polygenic variance that occurred when the QTL was added to a model with exclusively polygenic effects; responses = polygenic effects + errors.

### Data availability

All data utilized for both statistical and QTL mapping analyses in this study are provided in File S1.

## Results

### Spatial variability for Al saturation in the soil

**Figure 1:**
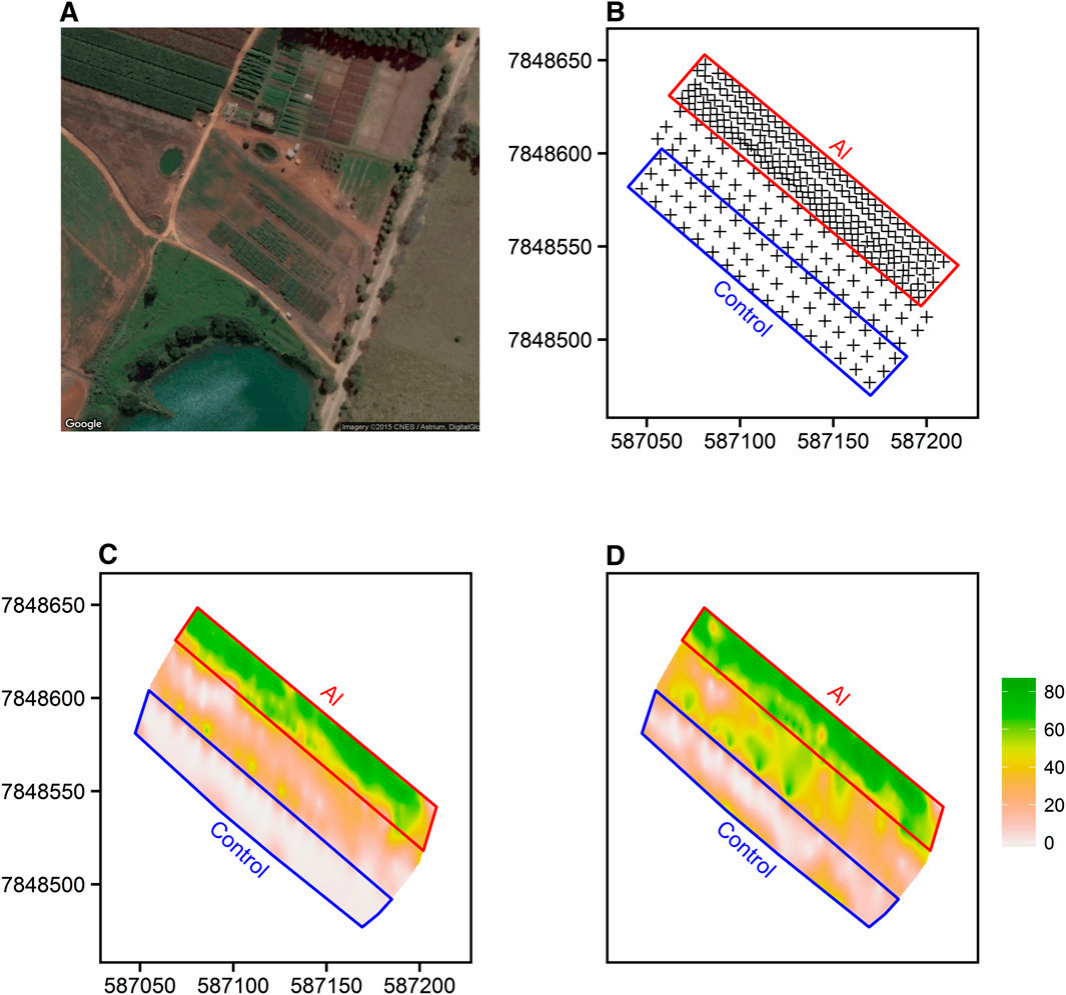
Field phenotyping sites. Satellite image (A) and respective soil sampling points (B). Al saturation (%) spatial variation in the superficial (0–20 cm) (C), and subsuperficial (20–40 cm) (D) soil layers. Adapted from Menezes *et al.* (2014). *x*- and *y*-axis in panels B, C and D correspond to spatial coordinates in Universal Transverse Mercator (UTM) 23k format. Satellite images were obtained on March 5, 2014.

Phenotyping sites were set up to assess Al tolerance in the field. Soil Al saturation was low, near 2% in the superficial layer (0–20 cm) in the control site, whereas much higher Al saturation was observed in the Al toxicity site, reaching 56% on average (Figure 1 and Table S2). The same large differences in Al saturation were observed in the subsoil (20–40 cm), with mean Al saturation values of approximately 15% and 65% for the control and Al toxicity sites, respectively. Spatial variability of soil pH is shown in Figure S1.

### Assessment of Al tolerance in the parents of the RIL population

**Figure 2:**
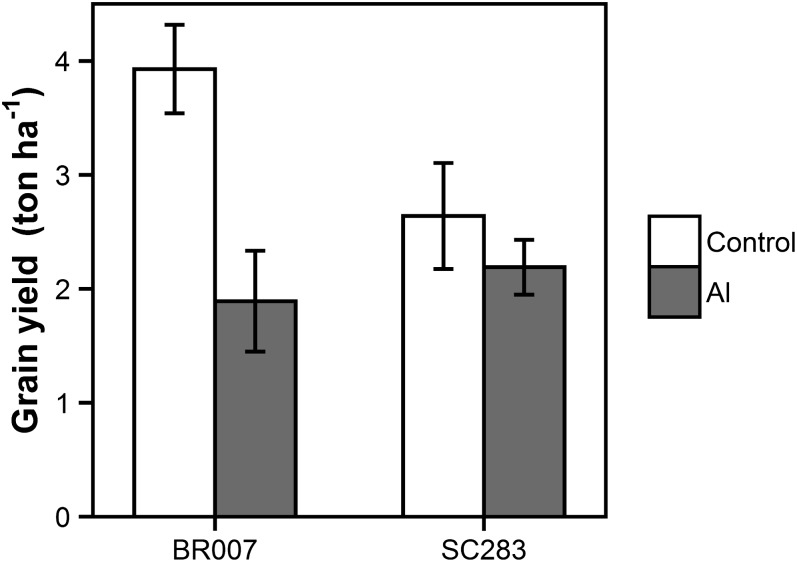
Phenotypic means for grain yield (ton ha^–1^) under control (2% Al saturation) and Al stress (56% Al saturation) conditions in the field for the parental lines BR007 and SC283. Means are based on 2-year trials. Least significant difference (LSD) = 0.59 (*P* = 0.05). Error bars are shown.

Average grain yield in the Al-sensitive parent of our RIL population, BR007, under control conditions was 3.9 ton ha^–1^, revealing a much higher yield potential compared to the Al-tolerant parent, SC283 (Figure 2). However, upon Al stress, grain yield for BR007 was approximately half of that in the control treatment, while SC283 was able to sustain yield under Al stress. Despite the higher yield potential for BR007 compared to SC283, under Al toxicity, grain yield in SC283 was higher compared to BR007.

### Genetic complexity of Al tolerance in the RIL population

The primary measure of Al tolerance in hydroponic media, relative net root growth (RNRG), was approximately 10-fold greater for SC283 than for BR007 on average (117% *vs.* 12%; Figure S2A), which was in agreement with previous studies indicating that SC283 is an important Al tolerance standard in sorghum (Duncan *et al.* 1983; Borgonovi *et al.* 1987; Furlani and Clark 1987; Furlani *et al.* 1991). In addition, much greater variation in RNRG was associated with the homozygous tolerant (TT) progeny compared to the homozygous sensitive (tt) progeny for *Alt_SB_*, suggesting the presence of minor Al tolerance loci distinct from *Alt_SB_* and/or the action of modifiers, with possible involvement in transgressive segregation for Al tolerance.

Compared to Al tolerance in hydroponics, transgressive segregation was much more evident for grain yield performance under high Al saturation, where segregants both below and above the mean of both parents were detected at a reasonable frequency (Figure S2, A and B). These results indicate that the genetic architecture underlying grain yield performance under Al toxicity is more complex compared to Al tolerance assessed in hydroponics.

The genetic variance for grain yield was larger in control compared to Al stress conditions in the field (Table S3), in agreement with previous reports indicating decrease of this variance component under abiotic stress conditions (Johnson and Frey 1967; Ud-Din *et al.* 1992; Kumar *et al.* 2007; Sabadin *et al.* 2012). Broad-sense heritability estimated for the RIL population was high, between 0.7 and 0.9, and it was highest for Al tolerance assessed in hydroponics under strict environmental control. Al toxicity reduced grain yield in the RIL population by approximately 30% and led to slightly lower heritability values compared to control conditions.

### Assessment of Al tolerance in isogenic hybrids

In order to assess the *Alt_SB_* effect in hybrids, we constructed isogenic hybrid stocks that contrasted for different *Alt_SB_* alleles within an otherwise uniform genetic background (Table S1). Analysis of variance of grain yield detected a significant environmental effect with high Al saturation causing a 23% reduction in grain yield (Table 1 and Table S4). Furthermore, this analysis emphasized a significant average female effect between ATF13A(tt) and ATF14A(TT), which was a striking 0.5 ton ha^–1^ (Table 1). This average effect arises from adding one copy of the Al tolerance allele, T, as tested across the four males to whom the female lines were crossed (Table S5). Because this average female effect includes comparisons both between 0 (tt), 1 (Tt), and 2 (TT) copies of the Al tolerance allele (Table S5), it also indicates there is advantage in breeding superior *Alt_SB_* alleles into both parents of hybrid stocks due to its additive gene action in field conditions.

**Table 1:** Means with respective least significant differences (*P* = 0.05) for environment and female main effects obtained by analysis of variance of eight isogenic hybrids grown in a split-plot design with four replications and a completely randomized design at the whole plot level (Table S4)

Average Effect		Grain Yield (ton ha^–1^)
Female	ATF13A	3.46
	ATF14A	3.96
	LSD (0.05)	0.45
Environment	Control	4.18
	Al	3.24
	LSD (0.05)	0.90

### Genetic architecture of Al tolerance in hydroponics and in the field

**Figure 3:**
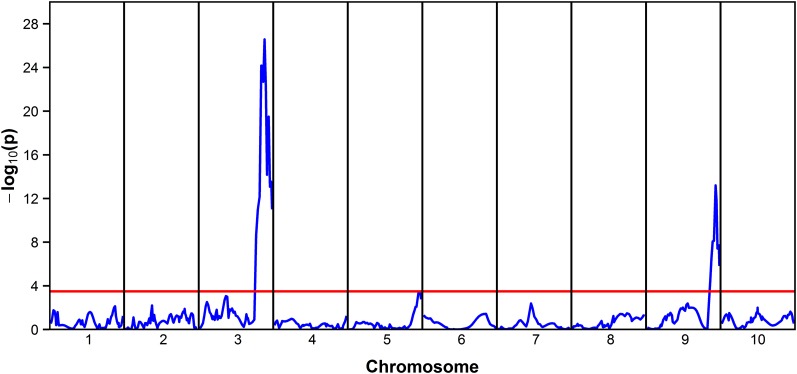
Graphical display of the QTL detected by multi-trait QTL analysis for relative net root growth (RNRG) after 5 d at {27} µM Al^3+^ in nutrient solution, and grain yield (ton ha^–1^) under 56% Al saturation stress in the field. The associated tail probability of the Wald statistics, *P*, is expressed as –log_10_(p), analogous to the usual LOD score profile. The red horizontal line indicates the significant threshold obtained using a correction for multiple testing following Li and Ji (2005), with a genome wide test level α = 0.05.

**Figure 4:**
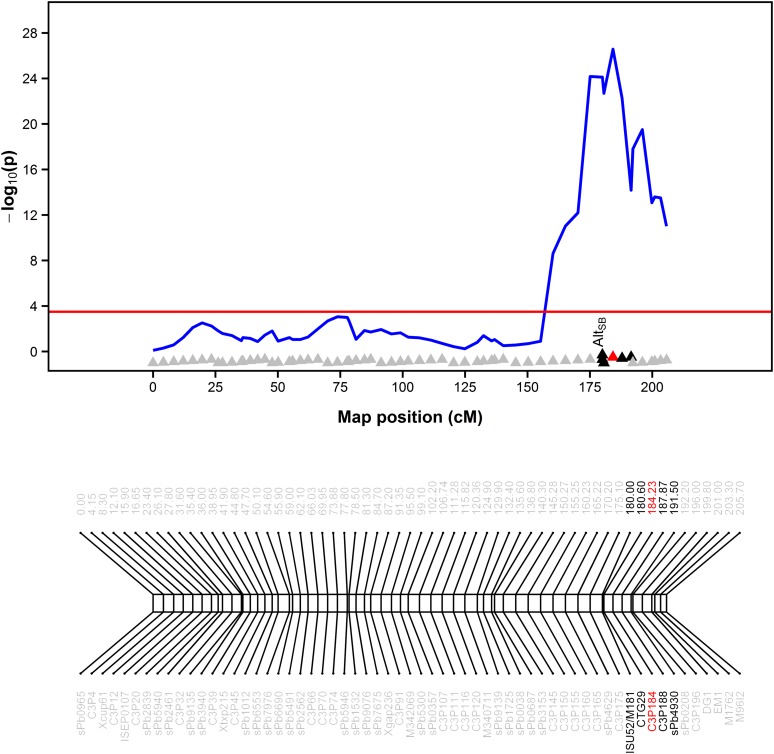
Detailed graphical display of the QTL detected by a multi-trait QTL analysis on chromosome 3 for relative net root growth (RNRG) after 5 d at {27} µM Al^3+^ in nutrient solution and grain yield (ton ha^–1^) under 56% Al saturation stress in the field. The associated tail probability of the Wald statistics, *P*, is expressed as –log_10_(p), analogous to the usual LOD score profile. The red horizontal line indicates the significant threshold obtained using the Li and Ji (2005) correction with α = 5%.

Multi-trait analysis revealed a single major QTL for Al tolerance on chromosome 3 assessed by quantifying either RNRG in hydroponics and grain yield in the field under high Al saturation (Figure 3). This QTL was detected at position 184.2 cM, tightly linked to the STS markers, CTG29 and M181 at positions 180.6 and 180 cM, respectively (Figure 4). The Al tolerance QTL assessed in hydroponics, *RNRG3*, explained half of the variance for RNRG, with the SC283 allele positively contributing to Al tolerance. Based on its position, *RNRG3/Gy3* is located in close proximity to *Alt_SB_* and *SbMATE* (Figure 4). As observed for *RNRG3*, the positive allele at *Gy3* was also donated by the Al-tolerant parent, SC283, indicating a pleiotropic action of the *Alt_SB_* locus on both sustained root elongation in nutrient solution, with an Al^3+^ activity of 27 µM and grain yield on soils with 56% Al saturation. *Gy3* was expressed both in control and Al stress conditions but the effect under Al toxicity was 0.6 ton ha^–1^, more than threefold greater than the effect estimated in control conditions (Table 2). In addition, the proportion of the phenotypic variation explained under low Al saturation was only 3.5%, contrasting with almost 38% under Al stress conditions.

**Table 2:** Estimates of QTL effects using multi-trait analysis for relative net root growth (log_10_ transformed ratio) and grain yield (ton ha^–1^)

Environment*^a^*	QTL	QTL Position (cM)*^b^*	Closest Markers	Effects	High Value Allele*^c^*	%GV	Avse
Hydroponics							
	*RNRG3*	184.2	ctg29 – sPb4930	0.71	SC283	50.10	(0.08)
	*RNRG9*	214.0	sPb1826 – sPb4087	0.34	SC283	11.30	(0.07)
				Total		**61.40**	
Field							
Control	*Gy3*	184.2	ctg29 – sPb4930	0.19	SC283	3.50	(0.09)
	*Gy9*	214.0	sPb1826 – sPb4087	0.66	BR007	44.00	(0.09)
				Total		47.50	
Al	*Gy3*	184.2	ctg29 – sPb4930	0.61	SC283	37.70	(0.10)
	*Gy9*	214.0	sPb1826 – sPb4087	0.36	BR007	12.70	(0.09)
				Total		**50.40**	

Average standard errors (Avse) for each effect are shown between parentheses. QTL are coded as relative net root growth (RNRG) or grain yield (Gy) numbered according to the chromosome where they were mapped. %GV stands for the percentage of the genetic variance that is explained by a given QTL (the percentage of the genetic variance explained after fitting all significant QTL in the model is shown in bold, following individual %GV estimates).

a Control: 2% soil aluminum saturation, Al: 56% soil aluminum saturation.

b QTL position corresponds to –log_10_(p) peak for a given QTL.

c Allele that increases the phenotype.

Our multi-trait analysis also detected a second Al tolerance QTL for both Al tolerance in hydroponics (*RNRG9*) and grain yield (*Gy9*) at position 214.0 cM on chromosome 9 (Figure 3), with *RNRG9* explaining a smaller proportion of the variation for Al tolerance in hydroponics (Table 2). As was the case for *RNRG3*, the positive allele at *RNRG9* was also donated by the Al-tolerant parent, SC283. In turn, the grain yield QTL, *Gy9*, explained a much larger portion of the genetic variance in control compared to Al toxicity conditions, and the allele increasing grain yield was donated by the Al-sensitive line, BR007, both under high and low Al saturation.

## Discussion

Our first step in linking hydroponics-based assessments of Al tolerance to a possible yield advantage elicited by the sorghum Al tolerance locus, *Alt_SB_*, was to design and set up field phenotyping sites where the effect of Al toxicity was as much as possible isolated from that caused by other abiotic stresses that occur jointly on acid soils (Bahia Filho *et al.* 1997). The Al saturation threshold in the field beyond which sorghum yield is reduced is around 20% Al saturation for sorghum growing in Oxisols/Ultisols (Gourley 1987). Based on that, the control (2% Al saturation) and Al toxicity (56% Al saturation) sites were designed to allow for the specific assessment of the impact of genetic determinants underlying Al tolerance on sorghum yield. Our field results for the parental lines supported previous hydroponics-based classifications of SC283 and BR007 as Al-tolerant and -sensitive lines (Magalhaes *et al.* 2004, 2007; Caniato *et al.* 2007, 2011, 2014), respectively, and that SC283 expresses Al tolerance in the field (Duncan *et al.* 1983; Gourley 1987; Furlani *et al.* 1991). A strong grain yield reduction caused by Al toxicity was observed for the Al-sensitive parent, BR007, showing that Al toxicity strongly overshadows the grain yield potential of Al-sensitive sorghum elite breeding lines.

Based on relative net root growth assessed in hydroponics, the Al tolerance QTL, *RNRG3*, and the grain yield QTL, *Gy3*, were tightly linked to the STS marker, CTG29, on chromosome 3. This marker is also tightly linked to *Alt_SB_* both genetically and physically (∼160 kb distant from *SbMATE*) and was used to previously positionally clone *SbMATE*, which underlies *Alt_SB_* (Magalhaes *et al.* 2007). The detailed genetic map of the *Alt_SB_* region is shown in Figure 4 and depicts CTG29 at position 180.6 cM on chromosome 3, while M181, which was the second flanking marker used to positionally clone *SbMATE* (and located in the same bacterial artificial chromosome as *SbMATE*) (Magalhaes *et al.* 2007), cosegregates with *Alt_SB_* at position 180 cM, along with the marker originally used to map *Alt_SB_*, ISU52 (Magalhaes *et al.* 2004). This indicates that *Alt_SB_* underlies both the *RNRG3* and *Gy3* QTL.

The alleles increasing phenotypic expression at *RNRG3* and *Gy3* were both donated by the Al-tolerant parent, SC283, and *Gy3* had a much higher phenotypic effect and explained a much larger proportion of the phenotypic variance under Al toxicity, compared to the control site with low Al saturation. From these results, a yield advantage of 0.6 ton ha^–1^ was determined, arising from substituting two copies of the BR007 allele with two copies of the Al tolerant (SC283) allele at *Alt_SB_*, which translates into a 26% grain yield increase under Al toxicity, based on the RIL grain yield mean.

Our studies with isogenic hybrid stocks indicate that *Alt_SB_* has an even stronger effect in hybrid stocks, with an on average 0.5 ton ha^–1^ female effect arising from one Al tolerance *Alt_SB_* allele. We also explicitly tested for additive *vs.* dominant gene action by introducing a covariate for the number of Al tolerance alleles (T) in the hybrids. We found the coefficient for this covariate to be the same as the average substitution effect estimated by fitting female and male in place of allele dosage (Table 1). Therefore, it is advantageous to introgress *Alt_SB_* into both parents due to its additive gene action on grain yield under Al toxicity. Overall, these results make Al tolerance conferred by *Alt_SB_* clearly the default trait when breeding for sorghum adaptation to acidic, Al toxic soils, via the development of homozygous or hybrid cultivars.

Although *Gy3* was expressed under low Al saturation, this QTL explained only 3.5% of the phenotypic variation under low Al saturation compared to 37.7% under high Al saturation, with a much lower effect of 0.19 ton ha^–1^ under low Al saturation. Recently, a sorghum genome-wide association mapping study conducted in West Africa, including gene-specific markers developed for *SbMATE*, revealed that *SbMATE* SNPs were highly associated with grain yield. Associations were found especially under low phosphorus (P) conditions in 29 sites in West Africa, explaining up to 16% of the genotypic variance (Leiser *et al.* 2014). Accordingly, this suggests a pleiotropic role of *SbMATE* in providing tolerance to two of the most serious abiotic stresses for sorghum in West Africa, Al toxicity and P deficiency. As discussed in the Leiser *et al.* (2014) study, overexpression of citrate synthesis and malate transporter genes in different species has resulted in both improved Al-tolerance and enhanced P uptake under low P conditions (Delhaize *et al.* 2009; Wang *et al.* 2012; Liang *et al.* 2013), supporting previous hypotheses that Al tolerance and P uptake can be conferred by similar mechanisms (Liao *et al.* 2006; Magalhaes *et al.* 2007). Therefore, because phosphorus binds tightly to Al and Fe oxides in the clay fraction of acid soils (Novais and Smyth 1999), it is possible that the comparatively mild phenotypic expression of *Gy3* under low Al saturation is due to enhancement of P acquisition, which could have been accompanied by a smaller Al tolerance effect due to somewhat higher Al saturation in the subsoil layer of the control site.

As an important intermediate in the Krebs cycle, citrate is essential for plant growth. Hence, the development and the use of root organic acid efflux to protect against Al stress could carry a significant energy cost for the plant, which may negatively affect plant yields (Liu *et al.* 2012). In support of this, in the absence of Al^3+^, roots of transgenic Arabidopsis lines overexpressing *AtALMT1* or *AtMATE* had rates of constitutive malate and citrate release that were two to ten times higher, respectively, than those of wild-type plants, and root growth was inhibited 20–30% compared with wild-type plants. This was attributed to the carbon loss from roots due to organic acid exudation, which exerted a measurable carbon cost to the plant (Liu *et al.* 2012). If such a penalty occurs in the case of *Alt_SB_*, where *SbMATE* expression is driven by its native promoter, the SC283 allele should decrease grain yield under control conditions. This might be expected because, in the absence of Al toxicity in the control site, the extremely low levels of *SbMATE* expression and citrate release that are inherent to the BR007 allele (Magalhaes *et al.* 2007; Melo *et al.* 2013) would result in minimal root loss of carbon, and thus yield advantage compared to the functional SC283 allele if significant citrate efflux occurred due to the SC283 allele under low Al saturation in the soil. As discussed above, the SC283 allele at *Gy3* actually slightly increased grain yield in the control site, demonstrating that no yield penalty arises from Al-induced citrate release elicited by the Al-activated citrate transporter, *SbMATE*. As such, *Alt_SB_*-based Al-tolerant cultivars developed for Al toxic soils should perform equally well in soils without Al toxicity, avoiding the time and the cost required to develop cultivars specific to different soil types.

This outcome is expected based on our current knowledge on physiological and molecular regulation of Al tolerance conferred by *SbMATE*. First, no substantial citrate release is observed in the absence of Al^3+^, in either Al-tolerant or Al-sensitive sorghum lines (Magalhaes *et al.* 2007; Melo *et al.* 2013), which may be due to another Al-binding protein that interacts with *SbMATE*, and is responsible for Al-activated root citrate efflux (Kochian *et al.* 2015). Second, although *SbMATE* is expressed in the absence of Al^3+^, its expression is localized to the root apex of Al tolerant near-isogenic lines carrying the SC283 allele compared to other root regions (Magalhaes *et al.* 2007), which is consistent with early reports of spatial localization of Al-induced organic acid release acting to protect Al sensitive zones specifically in the root apex (Ryan *et al.* 1993; Kochian *et al.* 2015). Moreover, using near-isogenic lines derived from BR007 and SC283, it has been recently shown that Al-induced *SbMATE* gene and protein expression is specifically localized to the epidermal and outer cortical cell layers of the root distal transition zone in the Al-resistant NIL (Sivaguru *et al.* 2013). This was shown to be the specific site of Al-induced root damage, and the time course of Al induction of *SbMATE* expression in the distal transition zone was precisely coincident with the onset of the recovery of this root region from Al-induced damage. Overall, the body of evidence underlying *SbMATE* function indicates that Al tolerance encoded by this sorghum MATE protein is under tight control to minimize the loss of carbon due to citrate release, preventing a yield penalty in the absence of Al toxicity.

The research findings presented here also uncovered a novel Al tolerance QTL on sorghum chromosome 9, which is consistent with previous reports of incomplete transfer of Al tolerance from donor lines to NILs based solely on *Alt_SB_* (Caniato *et al.* 2007, 2014). The Al tolerance and grain yield QTL, *RNRG9* and *Gy9*, were found at position 214 cM (confidence interval 207–221 cM) by multi-trait analysis, suggesting the presence of a single pleiotropic QTL affecting both traits. However, differently than *Gy3* and *RNRG3*, where positive alleles at both loci were donated by SC283, it was the SC283 allele that increased Al tolerance in hydroponics for *RNRG9* whereas the Al sensitive allele from BR007 at *Gy9* increased grain yield both in control and Al toxicity conditions in the soil. Interestingly, a major phenology-related QTL with a strong effect on grain yield has been previously found at position 218 cM on chromosome 9 in the same RIL population, with the positive allele also donated by the high-yielding parent, BR007 (Sabadin *et al.* 2012), as was the case for *Gy9* in the present study. This QTL is likely to correspond to *Sb-HT9.1* (Brown *et al.* 2008) as discussed in the Sabadin *et al.* (2012) study. Unlike *Gy3*, *Gy9* had a higher effect on grain yield and explained a much larger proportion of the genetic variance in control compared to Al stress conditions in the field, suggesting that *Gy9* is unspecific with regards to Al tolerance. Based on this, we speculate that *Gy9* and *RNRG9* could possibly be in fact distinct, with the former affecting primarily plant height/flowering time as described by Sabadin *et al.* (2012) while the allele donated by SC283 in *RNRG9* enhances Al tolerance assessed in hydroponics. Under this hypothesis, the lack of detection of an effect for *RNRG9* in terms of grain yield gain under Al toxicity in the field, with the positive allele coming from the Al-tolerant parent, SC283, may be accounted by an overshadowing effect of the likely phenology-related QTL, *Gy9*, or to an insufficient effect of *RNRG9* in providing grain yield advantage under Al toxicity.

For improving Al tolerance based on *Alt_SB_*, we have recently developed gene-specific markers associated with Al tolerance that allow not only for marker-assisted introgression but which are also amenable for large-scale allele mining approaches based on *Alt_SB_*, that can be used to identify accessions harboring *Alt_SB_* directly in the breeder´s gene pool, with obvious adaptive advantages (Caniato *et al.* 2014). Based on the findings reported here, molecular breeding based on *Alt_SB_* is of fundamental importance to improving sorghum grain yields on acid soils with Al toxicity. This should help to unlock food production potential in agricultural frontiers, with many of them ironically located where they are needed most for mankind, such as in the tropics and subtropics, where food security is most tenuous.

## Supplementary Material

Supporting Information
