# Supplementary material for: Back to Acid Soil Fields: The Citrate Transporter SbMATE Is a Major Asset for Sustainable Grain Yield for Sorghum Cultivated on Acid Soils
Source: G3 (Bethesda). 2015 Dec 17;6(2):475–84. doi: 10.1534/g3.115.025791 (PMC4751565; doi:10.1534/g3.115.025791)
Supplement: Supporting Information [file supp_6_2_475__index.html]

Back to Acid Soil Fields: The Citrate Transporter SbMATE Is a Major Asset for Sustainable Grain Yield for Sorghum Cultivated on Acid Soils — Supporting Information 

# Back to Acid Soil Fields: The Citrate Transporter SbMATE Is a Major Asset for Sustainable Grain Yield for Sorghum Cultivated on Acid Soils

## Supporting Information for Carvalho Jr. *et al.*, 2016

**Files in this Data Supplement:**

- Supporting Information - File contains supporting Figures and Tables. (.pdf, 349 KB)
- Figure S1 - Field phenotyping sites. (.pdf, 132 KB)
- Figure S2 - Frequency distribution for relative net root growth (RNRG) after five days at {27} µM Al3+ in nutrient solution (A) and grain yield (ton ha-1) under 56% Al saturation stress in the field (B) for the RIL population derived from the cross between SC283 (Al tolerant) and BR007 (Al sensitive). (.pdf, 98 KB)
- Table S1 - Genetic constitution of the isogenic hybrids, H1 to H8, along with the respective parents. (.pdf, 10 KB)
- Table S2 - Descriptive statistics for Al saturation (%) in the superficial (0 – 20 cm) and sub-superficial (20 – 40 cm) soil layers in the control and Al toxicity sites. (.pdf, 72 KB)
- Table S3 - Variance components, phenotypic means and heritability (*h*2) estimates are shown for relative net root growth (RNRG) assessed in hydroponics after five days of Al exposure and grain yield (ton ha-1) in control conditions and under high-Al saturation (Al) in the field.(.pdf, 113 KB)
- Table S4 - Analysis of variance with respective F probabilities of eight isogenic hybrids grown in a split-plot design with four replications and whole plots following a completely randomized design. (.pdf, 14 KB)
- Table S5 - Phenotypic means for grain yield (ton ha-1) under control (2% Al saturation) and Al stress (56% Al saturation) conditions in the field for the eight hybrids with distinct allelic combinations at the *AltSB* locus as depicted in Table S1.(.pdf, 12 KB)
- File S1 - Complete genotypic and phenotypic datasets. (.xlsx, 205 KB)
