# Supplementary material for: Back to Acid Soil Fields: The Citrate Transporter SbMATE Is a Major Asset for Sustainable Grain Yield for Sorghum Cultivated on Acid Soils
Source: G3 (Bethesda). 2015 Dec 17;6(2):475–84. doi: 10.1534/g3.115.025791 (PMC4751565; doi:10.1534/g3.115.025791)
Supplement: Supporting Information [file supp_g3.115.025791_025791SI.pdf]

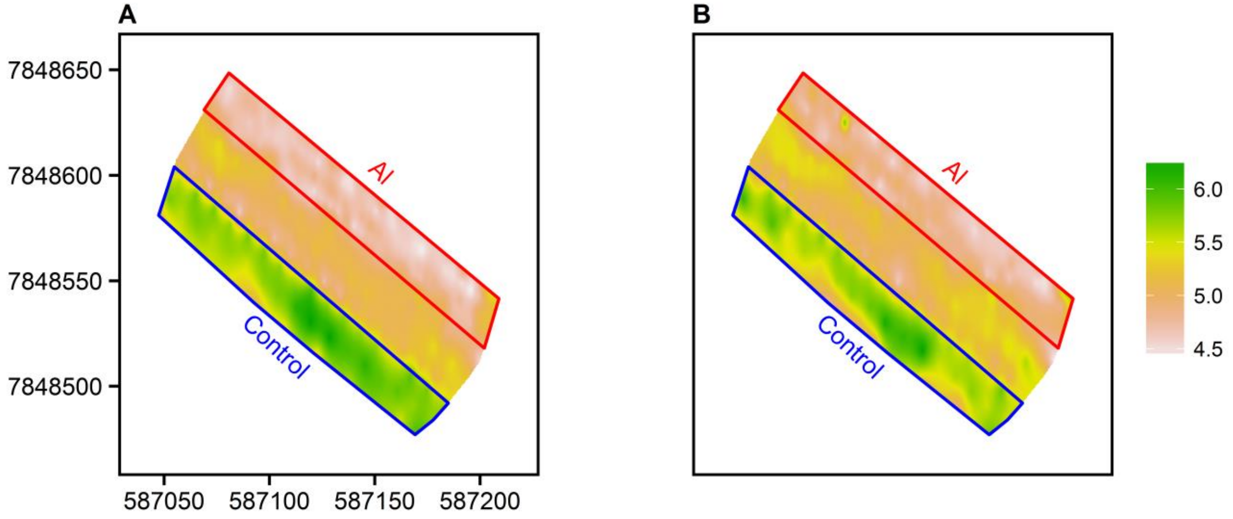

Figure S1 Field phenotyping sites. Spatial variation of soil pH in the superficial (0 – 20 cm) **(A)** and sub-superficial (20 – 40 cm) **(B)** soil layers. X- and Y-axis at panel's A, and B correspond to spatial coordinates in Universal Transverse Mercator (UTM) 23k format.

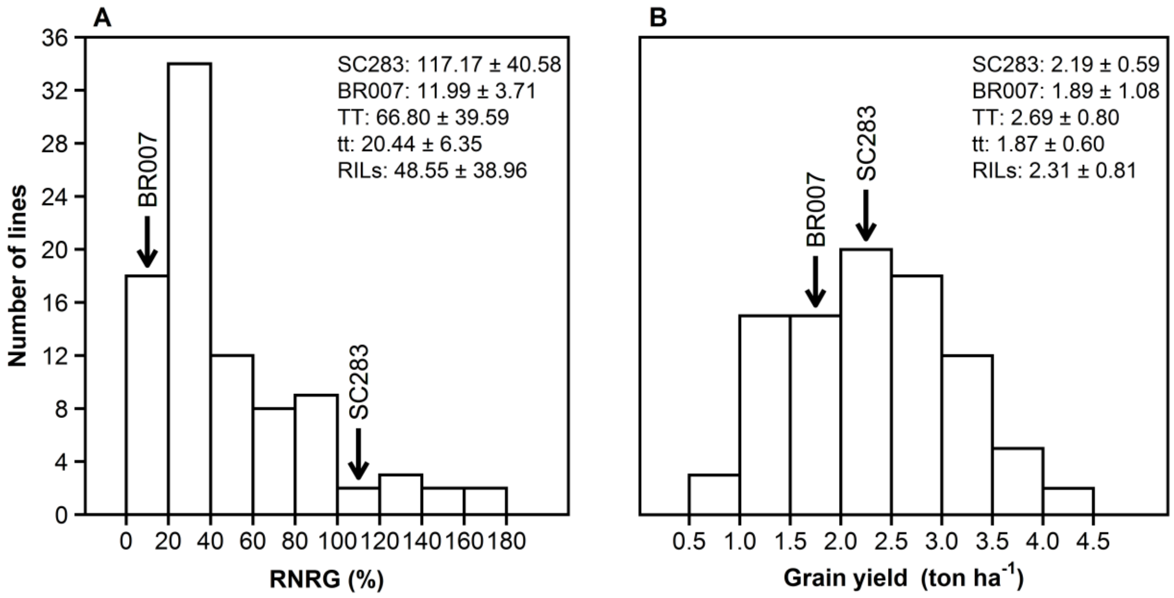

Figure S2 Frequency distribution for relative net root growth (RNRG) after five days at {27} µM Al^{3+} in nutrient solution **(A)** and grain yield (ton ha^{-1}) under 56% Al saturation stress in the field **(B)** for the RIL population derived from the cross between SC283 (Al tolerant) and BR007 (Al sensitive). Phenotypic means and standard deviations for the parents, homozygous tolerant (TT), homozygous sensitive (tt) progeny and the entire population (RILs) are shown.

Table S1 Genetic constitution of the isogenic hybrids, H1 to H8, along with the respective parents. The *Alt*$_{SB}$ genotypes, Al tolerant (TT), Al sensitive (tt) and heterozygous (Tt) are shown between parentheses.

		Male lines (R)			
Female lines (A)		BR012 (tt)	BR012(566) (TT)	BR012(SC549) (TT)	BR012(CMS225) (TT)
	ATF13A(tt)	H1(tt)	H3(Tt)	H5(Tt)	H7(Tt)
	ATF14A(TT)	H2(Tt)	H4(TT)	H6(TT)	H8(TT)

Table S2 Descriptive statistics for Al saturation (%) in the superficial (0 – 20 cm) and sub-superficial (20 – 40 cm) soil layers in the control and Al toxicity sites.

Al saturation (%)	Depth (cm)	Control	Al
Mean	0 – 20	2.1	55.9
	20 – 40	15.2	64.6
Median	0 – 20	0.4	60.3
	20 – 40	9.4	67.2
Standard deviation (SD)	0 – 20	5.0	18.4
	20 – 40	15.8	16.1

Table S3 Variance components, phenotypic means and heritability (h^2) estimates are shown for relative net root growth (RNRG) assessed in hydroponics after five days of Al exposure and grain yield (ton ha^{-1}) in control conditions and under high-Al saturation (Al) in the field.

Effects	RNRG	Grain yield (ton ha^{-1})	
		Control	Al
$\hat{\sigma}_g^2$	0.09	1.12	0.44
$\hat{\sigma}_e^2$	0.01	0.62	0.55
Mean	48.49	3.15	2.31
h^2	0.95	0.85	0.71

Table S4 Analysis of variance with respective F probabilities of eight isogenic hybrids grown in a split-plot design with four replications and whole plots following a completely randomized design. Treatment (Al versus control) was applied to whole plots, while the hybrids of the full factorial female x male crossing were allocated to the sub plots. Treatment main effects were tested at whole plot level, while all other effects were tested at subplot level.

Source of Variation	DF	SS	MS	VR	F Pr.
Environment	1	14.37	14.37	6.69*	0.04
Residual a	6	12.88	2.15	2.71	
Female	1	4.07	4.07	5.14*	0.03
Male	3	3.09	1.03	1.30	0.29
Environment*Female	1	0.04	0.04	0.04	0.83
Environment*Male	3	2.67	0.89	1.13	0.35
Female*Male	3	0.91	0.30	0.38	0.76
Environment*Female*Male	3	1.37	0.46	0.58	0.63
Residual b	42	33.25	0.79		

*Significant at 0.05 probability
DF: degrees of freedom
MS: mean square
SS: sum of squares
VR: variance ratio
F Pr.: probability (F-test).

Table S5 Phenotypic means for grain yield (ton ha^{-1}) under control (2% Al saturation) and Al stress (56% Al saturation) conditions in the field for the eight hybrids with distinct allelic combinations at the Alt_{SB} locus as depicted in Table S1.

		Male lines (R)			
Environment	Female lines (A)	BR012 (tt)	BR012(SC566) (TT)	BR012(SC549) (TT)	BR012(CMS225) (TT)
Control	ATF13A(tt)	3.93(tt)	4.20(Tt)	3.88(Tt)	3.80(Tt)
	ATF14A(TT)	4.25(Tt)	3.97(TT)	4.53(TT)	4.91(TT)
Al	ATF13A(tt)	2.21(tt)	3.31(Tt)	3.41(Tt)	2.91(Tt)
	ATF14A(TT)	2.94(Tt)	3.97(TT)	3.60(TT)	3.54(TT)
