# Supplementary material for: Back to Acid Soil Fields: The Citrate Transporter SbMATE Is a Major Asset for Sustainable Grain Yield for Sorghum Cultivated on Acid Soils
Source: G3 (Bethesda). 2015 Dec 17;6(2):475–84. doi: 10.1534/g3.115.025791 (PMC4751565; doi:10.1534/g3.115.025791)
Supplement: Supporting Information [file supp_g3.115.025791_FigureS1.pdf]

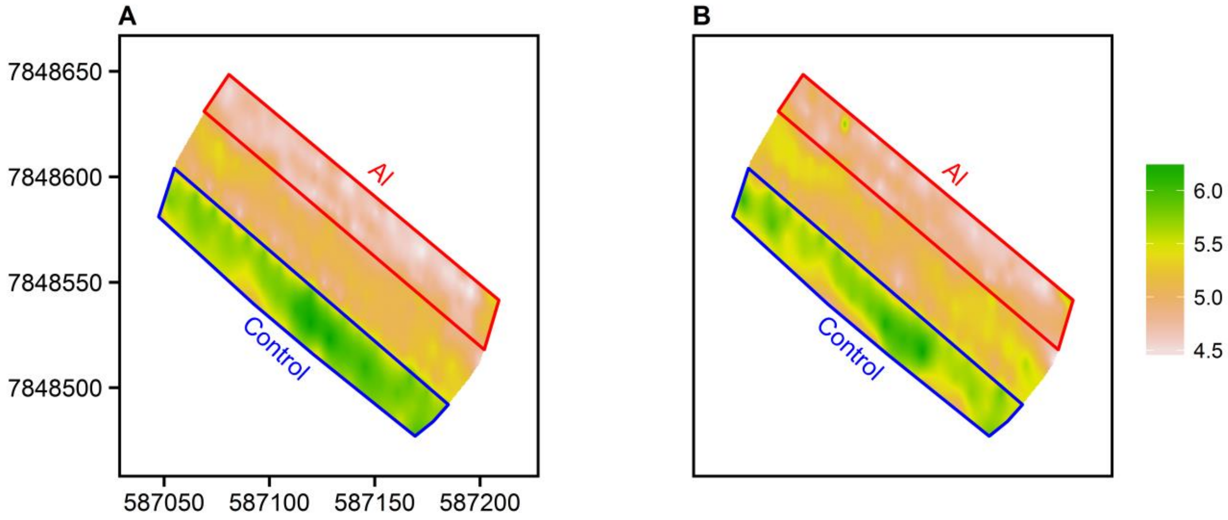

Figure S1 Field phenotyping sites. Spatial variation of soil pH in the superficial (0 – 20 cm) **(A)** and sub-superficial (20 – 40 cm) **(B)** soil layers. X- and Y-axis at panel's A, and B correspond to spatial coordinates in Universal Transverse Mercator (UTM) 23k format.
