# Supplementary material for: Back to Acid Soil Fields: The Citrate Transporter SbMATE Is a Major Asset for Sustainable Grain Yield for Sorghum Cultivated on Acid Soils
Source: G3 (Bethesda). 2015 Dec 17;6(2):475–84. doi: 10.1534/g3.115.025791 (PMC4751565; doi:10.1534/g3.115.025791)
Supplement: Supporting Information [file supp_g3.115.025791_FigureS2.pdf]

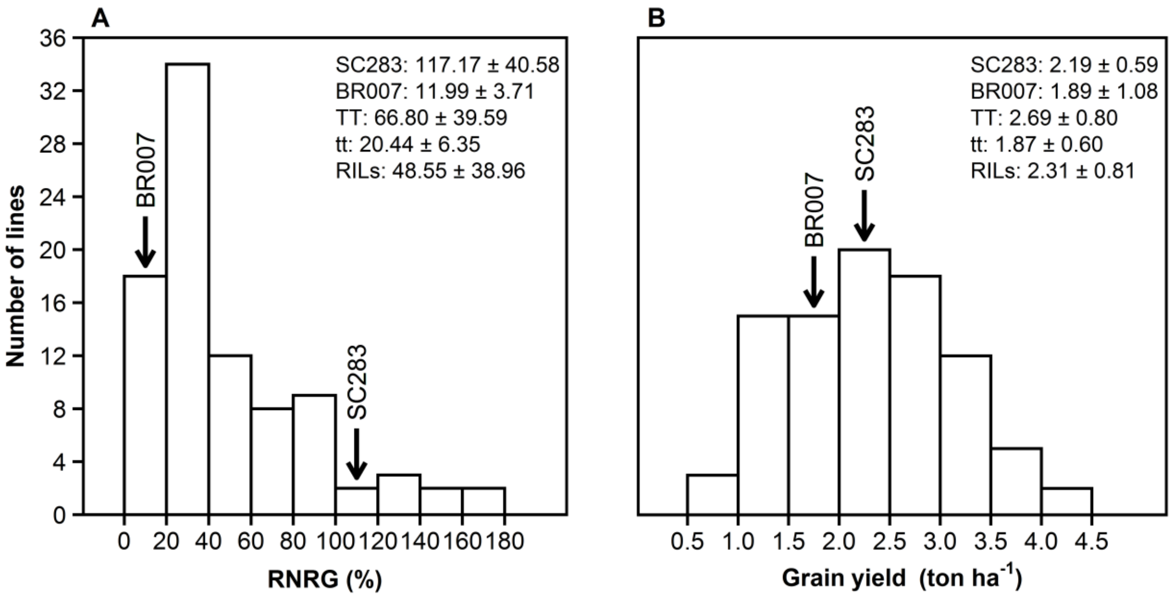

Figure S2 Frequency distribution for relative net root growth (RNRG) after five days at {27} µM Al^{3+} in nutrient solution **(A)** and grain yield (ton ha^{-1}) under 56% Al saturation stress in the field **(B)** for the RIL population derived from the cross between SC283 (Al tolerant) and BR007 (Al sensitive). Phenotypic means and standard deviations for the parents, homozygous tolerant (TT), homozygous sensitive (tt) progeny and the entire population (RILs) are shown.
