# Supplementary material for: Back to Acid Soil Fields: The Citrate Transporter SbMATE Is a Major Asset for Sustainable Grain Yield for Sorghum Cultivated on Acid Soils
Source: G3 (Bethesda). 2015 Dec 17;6(2):475–84. doi: 10.1534/g3.115.025791 (PMC4751565; doi:10.1534/g3.115.025791)
Supplement: Supporting Information [file supp_g3.115.025791_TableS1.pdf]

Table S1 Genetic constitution of the isogenic hybrids, H1 to H8, along with the respective parents. The *Alt*$_{SB}$ genotypes, Al tolerant (TT), Al sensitive (tt) and heterozygous (Tt) are shown between parentheses.

		Male lines (R)			
		BR012 (tt)	BR012(566) (TT)	BR012(SC549) (TT)	BR012(CMS225) (TT)
Female lines (A)	ATF13A(tt)	H1(tt)	H3(Tt)	H5(Tt)	H7(Tt)
	ATF14A(TT)	H2(Tt)	H4(TT)	H6(TT)	H8(TT)
