# Supplementary material for: Back to Acid Soil Fields: The Citrate Transporter SbMATE Is a Major Asset for Sustainable Grain Yield for Sorghum Cultivated on Acid Soils
Source: G3 (Bethesda). 2015 Dec 17;6(2):475–84. doi: 10.1534/g3.115.025791 (PMC4751565; doi:10.1534/g3.115.025791)
Supplement: Supporting Information [file supp_g3.115.025791_TableS2.pdf]

Table S2 Descriptive statistics for Al saturation (%) in the superficial (0 – 20 cm) and sub-superficial (20 – 40 cm) soil layers in the control and Al toxicity sites.

Al saturation (%)	Depth (cm)	Control	Al
Mean	0 – 20	2.1	55.9
	20 – 40	15.2	64.6
Median	0 – 20	0.4	60.3
	20 – 40	9.4	67.2
Standard deviation (SD)	0 – 20	5.0	18.4
	20 – 40	15.8	16.1
