# Supplementary material for: Back to Acid Soil Fields: The Citrate Transporter SbMATE Is a Major Asset for Sustainable Grain Yield for Sorghum Cultivated on Acid Soils
Source: G3 (Bethesda). 2015 Dec 17;6(2):475–84. doi: 10.1534/g3.115.025791 (PMC4751565; doi:10.1534/g3.115.025791)
Supplement: Supporting Information [file supp_g3.115.025791_TableS3.pdf]

Table S3 Variance components, phenotypic means and heritability (h^2) estimates are shown for relative net root growth (RNRG) assessed in hydroponics after five days of Al exposure and grain yield (ton ha^{-1}) in control conditions and under high-Al saturation (Al) in the field.

Effects	RNRG	Grain yield (ton ha^{-1})	
		Control	Al
$\hat{\sigma}_g^2$	0.09	1.12	0.44
$\hat{\sigma}_e^2$	0.01	0.62	0.55
Mean	48.49	3.15	2.31
h^2	0.95	0.85	0.71
