# Supplementary material for: Back to Acid Soil Fields: The Citrate Transporter SbMATE Is a Major Asset for Sustainable Grain Yield for Sorghum Cultivated on Acid Soils
Source: G3 (Bethesda). 2015 Dec 17;6(2):475–84. doi: 10.1534/g3.115.025791 (PMC4751565; doi:10.1534/g3.115.025791)
Supplement: Supporting Information [file supp_g3.115.025791_TableS4.pdf]

Table S4 Analysis of variance with respective F probabilities of eight isogenic hybrids grown in a split-plot design with four replications and whole plots following a completely randomized design. Treatment (Al versus control) was applied to whole plots, while the hybrids of the full factorial female x male crossing were allocated to the sub plots. Treatment main effects were tested at whole plot level, while all other effects were tested at subplot level.

Source of Variation	DF	SS	MS	VR	F Pr.
Environment	1	14.37	14.37	6.69*	0.04
Residual a	6	12.88	2.15	2.71	
Female	1	4.07	4.07	5.14*	0.03
Male	3	3.09	1.03	1.30	0.29
Environment*Female	1	0.04	0.04	0.04	0.83
Environment*Male	3	2.67	0.89	1.13	0.35
Female*Male	3	0.91	0.30	0.38	0.76
Environment*Female*Male	3	1.37	0.46	0.58	0.63
Residual b	42	33.25	0.79		

*Significant at 0.05 probability
DF: degrees of freedom
MS: mean square
SS: sum of squares
VR: variance ratio
F Pr.: probability (F-test).
