# Supplementary material for: Back to Acid Soil Fields: The Citrate Transporter SbMATE Is a Major Asset for Sustainable Grain Yield for Sorghum Cultivated on Acid Soils
Source: G3 (Bethesda). 2015 Dec 17;6(2):475–84. doi: 10.1534/g3.115.025791 (PMC4751565; doi:10.1534/g3.115.025791)
Supplement: Supporting Information [file supp_g3.115.025791_TableS5.pdf]

Table S5 Phenotypic means for grain yield (ton ha^{-1}) under control (2% Al saturation) and Al stress (56% Al saturation) conditions in the field for the eight hybrids with distinct allelic combinations at the Alt_{SB} locus as depicted in Table S1.

		Male lines (R)			
Environment	Female lines (A)	BR012 (tt)	BR012(SC566) (TT)	BR012(SC549) (TT)	BR012(CMS225) (TT)
Control	ATF13A(tt)	3.93(tt)	4.20(Tt)	3.88(Tt)	3.80(Tt)
	ATF14A(TT)	4.25(Tt)	3.97(TT)	4.53(TT)	4.91(TT)
Al	ATF13A(tt)	2.21(tt)	3.31(Tt)	3.41(Tt)	2.91(Tt)
	ATF14A(TT)	2.94(Tt)	3.97(TT)	3.60(TT)	3.54(TT)
